# Quantitative evaluation of PET respiratory motion correction using real- time PET/MR simulated data

**DOI:** 10.1186/2197-7364-1-S1-A62

**Published:** 2014-07-29

**Authors:** Irene Polycarpou, Charalampos Tsoumpas, Andrew King, Paul K Marsden

**Affiliations:** Division of Imaging Sciences and Biomedical Engineering, King’s College London, London, UK; Division of Medical Physics, University of Leeds, Leeds, UK

The impact of respiratory motion correction on quantitative accuracy in PET imaging is evaluated using real-time simulations for variable patient specific characteristics such as tumor malignancy and respiratory pattern. Respiratory patterns from real patients were acquired, with long quiescent motion periods (type-1) as commonly observed in most of the patients and with long term amplitude variability as it is expected under conditions of difficult breathing (type-2). The respiratory patterns were combined with an MR-derived motion model to simulate real-time 4D PET/MR datasets. Lung and liver tumors were simulated with diameters ranging of 10 and 12 mm and tumor to background ratio ranging from 3:1 to 6:1. Projection data for 6 and 3 mm PET resolution were generated for Philips Gemini scanner and reconstructed without and with motion correction using OSEM (2 iterations, 23 subsets). Motion correction was incorporated into the reconstruction process based on MR-derived motion fields. Tumors peak standardized uptake values (SUVpeak) were calculated from thirty noise realizations. Respiratory motion correction improves the quantitative performance with the greatest benefit observed for patients of breathing type-2. For breathing type-1 after applying motion correction SUVpeak of 12 mm liver tumor with 6:1 contrast was increased by 46% for a current PET resolution (i.e. 6 mm) and 47% for a higher PET resolution (i.e. 3 mm). Furthermore, the benefit of higher scanner resolution is small for torso imaging unless motion correction is applied. In particular, for large liver tumor (12 mm) with low contrast (3:1) after motion correction the SUVpeak was 34% increased for 6 mm resolution and 50% increased for a higher PET resolution (i.e. 3 mm resolution. This investigation indicates high impact of respiratory motion correction on tumor quantitative accuracy and its importance in order to benefit from the increased resolution of future PET scanners.

